# A Novel Digital Care Management Platform to Monitor Clinical and Subclinical Disease Activity in Multiple Sclerosis

**DOI:** 10.3390/brainsci11091171

**Published:** 2021-09-03

**Authors:** Wim Van Hecke, Lars Costers, Annabel Descamps, Annemie Ribbens, Guy Nagels, Dirk Smeets, Diana M. Sima

**Affiliations:** 1 icometrix, 3012 Leuven, Belgium; lars.costers@icometrix.com (L.C.); annabel.descamps@icometrix.com (A.D.); annemie.ribbens@icometrix.com (A.R.); guy.nagels@vub.be (G.N.); dirk.smeets@icometrix.com (D.S.); diana.sima@icometrix.com (D.M.S.); 2 AI Supported Modelling in Clinical Sciences (AIMS), Vrije Universiteit Brussel, 1050 Brussels, Belgium; 3 Department of Engineering, University of Oxford, Oxford OX1 3PJ, UK

**Keywords:** multiple sclerosis, ico**mpanion**, ico**brain**, eHealth, digital health technology, mobile application, patient reported outcomes, magnetic resonance imaging

## Abstract

In multiple sclerosis (MS), the early detection of disease activity or progression is key to inform treatment changes and could be supported by digital tools. We present a novel CE-marked and FDA-cleared digital care management platform consisting of (1) a patient phone/web application and healthcare professional portal (ico**mpanion**) including validated symptom, disability, cognition, and fatigue patient-reported outcomes; and (2) clinical brain magnetic resonance imaging (MRI) quantifications (ico**brain ms**). We validate both tools using their ability to detect (sub)clinical disease activity (known-groups validity) and real-world data insights. Surveys showed that 95.6% of people with MS (PwMS) were interested in using an MS app, and 98.2% were interested in knowing about MRI changes. The ico**mpanion** measures of disability (*p* < 0.001) and symptoms (*p* = 0.005) and ico**brain ms** MRI parameters were sensitive to (sub)clinical differences between MS subtypes. ico**brain ms** also decreased intra- and inter-rater lesion count variability and increased sensitivity for detecting disease activity/progression from 24% to 76% compared to standard radiological reading. This evidence shows PwMS’ interest, the digital care platform’s potential to improve the detection of (sub)clinical disease activity and care management, and the feasibility of linking different digital tools into one overarching MS care pathway.

## 1. Introduction

Today, more than 2.8 million people are living with multiple sclerosis (MS), making it the most common progressive neurological condition in young people [1]. MS is characterized either by periods of relapses and remission or a progressive disability pattern. Currently, there are over 20 disease-modifying treatments (DMTs) available, aiming to slow down relapses and disease progression [2]. Thanks to these DMTs, the health of people with MS (PwMS), expressed in quality-adjusted life years (QALYs), has been estimated to have increased by 66% since the launch of the first drug in 1993 [3]. However, despite the increased availability of DMTs, 26% to 40% of PwMS are estimated to be on a suboptimal treatment [4,5].

These findings illustrate the challenge in MS care, which is providing individual PwMS with the right drug at the right time. Hence, in order to make informed treatment decisions, it is crucial to measure disease activity and progression in a standardized manner. In this context, disease activity and progression are typically evaluated by the clinical assessment of relapses and disability (measured by the Expanded Disability Status Scale (EDSS)), and longitudinal changes on the brain magnetic resonance imaging (MRI) scans (looking at new/enlarging lesions and/or brain atrophy) [6].

However, it is known that clinical disease activity and progression often go unnoticed during clinical assessment, partly due to the problem that relapses are systematically underreported by around half of PwMS [6,7]. In addition, MS progression goes beyond relapses and physical disability worsening, as problems with memory but also linguistic and verbal fluency problems are known to be important components of MS-related disability [8,9]. These components are often not routinely assessed during the patient visit or are based on the PwMS’ recollection on how they have been doing since the last visit. In addition, it has been demonstrated that there is a significant clinician-dependent variability in the assessment of MS patient disease activity [10].

The second component of evaluating disease activity is based on the assessment of subclinical progression on brain MRI scans. International guidelines recommend the acquisition of brain (and increasingly spinal cord) MRI scans for diagnosis and a yearly scan for follow-up [11]. However, in a clinical setting, brain MRI reading is known to be qualitative, based on a visual assessment, and radiologist-dependent, leading to discrepancies in the radiological reports [12]. Indeed, it has been reported that up to 24% of brain MRI reporting contains discrepancies when reviewed by a panel of radiologists [12].

There is great promise in implementing digital health solutions to standardize MS care, as they can improve efficiency and workflow, and complement clinical expertise. A recent study indicated healthcare professionals’ (HCPs) most crucial problems in MS disease management to be the lack of forwarding of information by the patient, the need for the patient to visit on site for inquiries and poor reachability of PwMS, for which digital telemonitoring tools can be a solution [13].

Remote patient monitoring through medical health (mHealth) applications in MS care allows for a more continuous and data-driven monitoring of symptoms and disease progression [14,15]. Regular standardized check-ins through mHealth apps have the potential to mitigate the underreporting of important clinical events [7] and bridge the information gap between annual neurology visits. It has been estimated that personalized medicine tools in MS have the potential to increase the impact of treatments by more than 50% by quantifying both disease activity (clinical and subclinical) and the risk of side effects [3]. In addition to the value of mHealth tools for health-care professionals (HCPs), there is also the potential to further empower PwMS, resulting in an increased self-management and allowing more open and early conversations about disease progression [16,17].

In addition to mHealth applications, artificial intelligence (AI) solutions have been developed to detect and quantify disease activity on MRI scans, which play a central role in disease management. In the last decades, several software tools have been developed and applied for research and clinical trials. Examples of widely used neuroimage analysis packages for research purposes include Freesurfer (https://surfer.nmr.mgh.harvard.edu accessed on 27 August 2021), FMRIB Software Library (FSL; https//fsl.fmrib.ox.ac.uk/fsl, accessed on 27 August 2021), and Statistical Parametric Mapping (SPM; https://www.fil.ion.ucl.ac.uk/spm, accessed on 27 August 2021). However, only very few brain MRI solutions exist that have been thoroughly validated and cleared as a medical device for clinical use [18].

In this paper, we present a novel digital care management platform for MS that aims at standardizing MS patient care and allowing more data-driven clinical decisions in the MS care pathway. The platform includes a CE marked and FDA cleared mHealth application that collects patient-reported information in the period of time between neurology visits, a CE marked and FDA cleared solution that quantifies clinically relevant brain MRI changes in PwMS, and the necessary software solutions that guarantee a seamless integration of the digital MS care management platform into the clinical workflow. We investigate the needs and interests of PwMS concerning such solutions, and their potential to improve the detection of (sub-)clinical disease activity and care management of MS.

## 2. Materials and Methods

### 2.1. MS Care Management Platform

**Figure 1:**
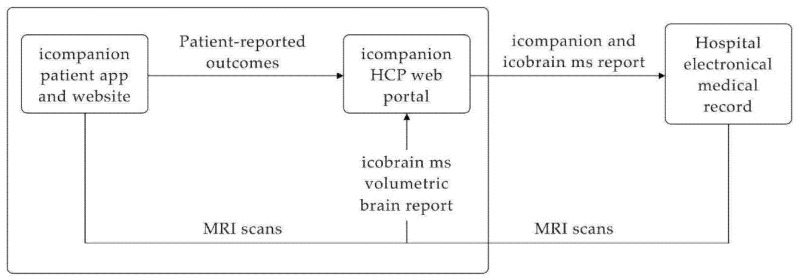
The MS care management platform consists of the ico**mpanion** patient app and website, ico**mpanion** HCP web portal, integration with ico**brain ms** volumetric brain reports and integration with hospital’s electronic medical records.

As illustrated in Figure 1, the care management platform consists of multiple components: (1) the ico**mpanion** patient mobile phone application (available on Android and iOS) and website (accessible via web browser: icompanion.ms), (2) the ico**mpanion** web portal for HCPs (accessible via web browser), (3) the ico**brain ms** volumetric brain reports and (4) integrations with hospitals’ Picture Archiving and Communication System (PACS) and electronic medical record (EMR) systems.

Both ico**mpanion** and ico**brain ms** are registered medical devices and were developed by ico**metrix** (Leuven, Belgium). According to FDA regulation, ico**mpanion** is a class 1 medical device, and under EU MDD regulation a class 1 medical device. ico**brain ms** is an FDA class 2 medical device and MDD class 1m medical device. ico**metrix**’ secure cloud is ISO13485 and ISO27001 certified and GDPR and HIPAA compliant regarding the Security and Privacy Rules. Incoming DICOM files of MRI scans are pseudonymized according to HIPAA standard and all fields containing private patient information are removed, except patient gender and birth date (transformed to YYYY-01-01) in order to be able to provide a correct analysis and compare the patient with a healthy population.

#### 2.1.1. icom**panion** Patient App and Website

**Figure 2:**
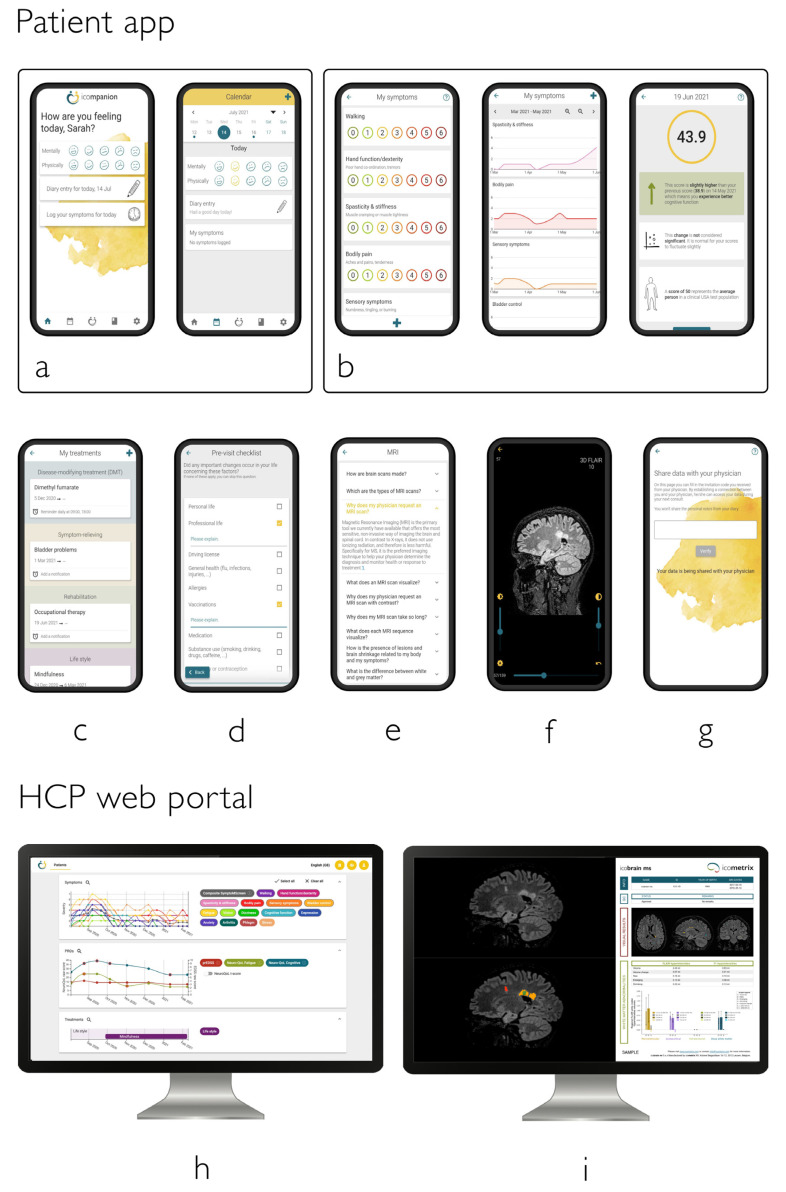
Overview of the ico**mpanion** patient app (**a**–**g**) and HCP platform features (**h**,**i**): (**a**) easy check-in and diary, (**b**) PROs for symptoms, EDSS, fatigue, cognition, …, (**c**) treatment logging and reminders, (**d**) preparation neurologist visit, (**e**) knowledge center, (**f**) MRI viewer, (**g**) linking with MS team, (**h**) interactive overview of PRO data and downloadable reports and (**i**) automatic import of MRI scans from hospital PACS system and integration with ico**brain ms** reports.

Using the ico**mpanion** app and website, PwMS can keep a diary, log symptoms, and perform tests for body function, cognitive function, and fatigue (Figure 2) based on clinically validated patient reported outcomes (PROs) described below [19,20,21,22], which can be shared with the patient’s clinical team. In addition, PwMS can add treatment information, from DMTs to symptomatic and rehabilitation treatments, and set reminders on when to take or perform their treatment. Furthermore, PwMS can easily upload their MRIs (via the patient website) and view them (via patient website and app) as well as learn about topics related to MS (e.g., MS types, MRI, lesions). Finally, PwMS can prepare their consultations using a pre-visit checklist, the answers of which are also shared with the patient’s clinical team (e.g., ‘Do you need any new prescription, certificates or reimbursement documents?’).

The clinically validated PROs included into ico**mpanion** are the SymptoMScreen, a patient-reported Expanded Disability Status Scale (prEDSS), Neuro-QoL (V1.0) Fatigue short-form, and the Neuro-QoL (V2.0) Cognitive Function short-form:

- The SymptoMScreen [21] is a 12-item battery for MS-related symptoms with a 7-point Likert scale per functional domain. Scores range from ‘not affected at all’ (score = 0) to ‘total limitation’ (score = 6). The SymptoMScreen composite is a score that summarizes general symptom severity by summing up the entered twelve symptom severities.
- The prEDSS is a patient-reported version of the Expanded Disability Status Scale (EDSS) which has shown to correlate well (Pearson’s coefficient 0.85) with a neurologist-scored EDSS [22].
- The Neuro-QoL (V1.0) Fatigue and (V2.0) Cognitive short-forms are short questionnaires consisting of eight items scored on a five-point scale each [23], and have been clinically validated in MS [19,20]. The Neuro-QoL t-scores reported in this paper are standardized scores with a mean of 50 and standard deviation (SD) of 10 compared to a reference population (for Cognitive a US general reference sample, for Fatigue a clinical US sample; http://www.healthmeasures.net/images/neuro_qol/Neuro_QOL_Scoring_Manual_Mar2015.pdf accessed on 27 August 2021). As an example, a t-score of 60 means that a person scores 1 SD higher than the reference sample.

#### 2.1.2. ico**brain ms** Volumetric MRI Analyses

**Figure 3:**
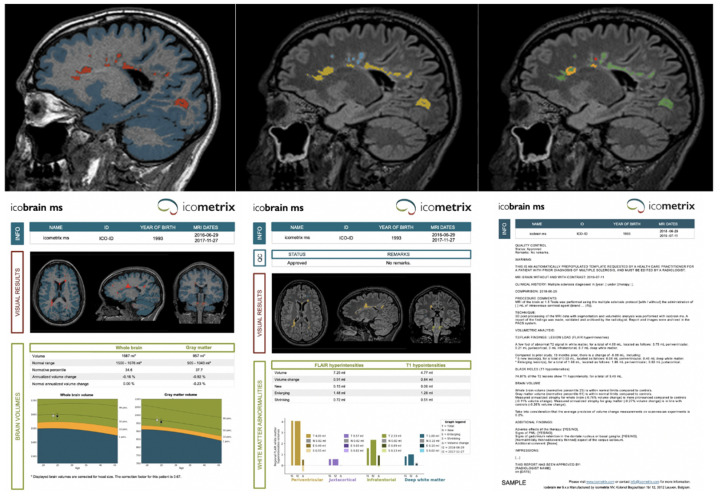
Output of the ico**brain ms** software, which is integrated with the local PACS. In the top row, a sagittal slice of the annotated images (which are presented in the same space as the original scans) is shown. The bottom row includes the quantitative ico**brain ms** reports and an example of the pre-populated radiological report.

ico**brain ms** is an AI software solution for brain magnetic resonance image analysis in MS, producing annotated images and pre-populated radiological reports (icometrix.com/services/icobrain-ms accessed on 27 August 2021). The main components of ico**brain ms** are the brain tissue segmentation and MS lesion segmentation on single-time point T1-weighted and FLAIR scans, as well as specific longitudinal volume change computations for establishing brain atrophy rates and lesion evolution. Whole brain and gray matter volumes, normalized for head size, are compared against age- and sex-matched reference controls populations. In Figure 3, an example of the ico**brain ms** output is shown, including the annotated images, the quantitative reports, and the pre-populated structured radiological report that are provided in the local PACS system and available by the time the radiological reading starts. In the top row of Figure 3, a sagittal slice of the annotated images (which are presented in the same space as the original scans) is shown, the bottom row includes the quantitative ico**brain ms** reports and an example of the pre-populated radiological report:

- top left = T1 overlaid with gray matter segmentation (blue) and T1 lesions (red);
- top middle = FLAIR overlaid with lesion segmentations color coded by location: periventricular (yellow), deep white matter (blue), juxtacortical (purple), and infratentorial (green);
- top right = FLAIR overlaid with color coded lesion changes compared to last available (or selected) scan: existing (green), enlarging (orange), new (red) lesions.
- bottom left = the quantitative report of whole brain and gray matter volume and atrophy (and comparison with healthy population).
- bottom middle = the quantitative report of existing, enlarging and, new FLAIR lesions and their location.
- bottom right = an example of a pre-populated radiological structured template, which already includes the ico**brain ms** measures and is available in the local language.

The technical details, validation, and clinical usefulness of the methodology have been published previously [24,25,26,27,28]. The software is seamlessly integrated in the clinical workflow via ico**bridge** (see Section 2.1.3 or https://icobridge.icometrix.com accessed on 27 August 2021). It includes direct and secure upload from the hospital’s image archiving system to ico**metrix**’ servers, where the AI pipeline is run, and secure transfer of reports and annotated images back to the hospital’s system in time for the radiological reading.

#### 2.1.3. ico**mpanion** HCP Portal

In the HCP portal, HCPs can access the entered ico**mpanion** PRO data from their linked PwMS as well as their MRI images and ico**brain ms** volumetric brain reports (Figure 1, Figure 2 and Figure 3). Access to the data on this portal can be easily managed via the MS team functionality, which allows the set-up of a care team with different team members and roles. The entered ico**mpanion** PRO data can be viewed in an interactive plot with tools to help the interpretation, and HCPs can download pdf reports. HCPs can also view the PwMS’ uploaded MRIs, and using ico**bridge** (https://icobridge.icometrix.com accessed on 27 August 2021), ico**metrix**’ secure DICOM gateway, MRI scans can also be automatically imported from a hospital’s PACS system. All imported MRI scans are analyzed using the ico**brain ms** volumetric analysis, after which a report can be downloaded from the HCP portal (Figure 3). The ico**mpanion** and ico**brain ms** reports (and raw data points or intermediate results) can be imported into a hospital’s EMR system. Using the HL7 v2 protocol, these reports can be sent over to the EMR, either in their native pdf format, coded values or as simple text. From there on this data can for instance be shown in a radiologic report or attached to a study for future reference.

### 2.2. Patient Perspective

#### 2.2.1. Patient Survey 1: Telemonitoring Tools for Monitoring Clinical Disease Activity

In order to develop a patient monitoring tool that responds to the needs of PwMS and fits PwMS’ everyday life, a survey was sent out to PwMS with MS via local patient support groups without any randomization. In this survey, answered by 45 PwMS, the PwMS were asked about their knowledge about important topics such as MRI, EDSS, etc., which was used to develop educational content for ico**mpanion**. Next, the PwMS were asked about their attitude towards an app to monitor MS, different possible features, and their interest in using such an app.

#### 2.2.2. Patient Survey 2: MR Imaging for Monitoring Subclinical Disease Activity

Together with iConquerMS (iConquerMS.org), a survey was sent out in June 2020 [29], with questions about MRI access, viewing and knowledge, which was answered by 876 PwMS. As an example, questions included (see Appendix A and Appendix B for the complete list of questions and possible answers in the survey):

- ‘Would you like to view your MRI images on your own—Why or why not?’
- ‘If you have or had access to your own MRIs, would you be interested in knowing whether there were any changes between one MRI and the next (such as new lesions or loss of brain volume)?’

### 2.3. Monitoring Clinical Disease Activity Using ico**mpanion**: Real-World Evidence

#### 2.3.1. Study Synopsis-Characterizing MS Types with ico**mpanion**

The ico**mpanion** app and website were launched in July 2020. We describe the information entered into ico**mpanion** and investigate the validity of the real-world collected PRO data by looking at their sensitivity to clinical differences between MS types based on known-groups validity. These MS types include clinically isolated syndrome (CIS), relapsing-remitting MS (RRMS), secondary progressive MS (SPMS), and primary progressive MS (PPMS). The descriptions of the collected data and the analyses were based on an anonymized dataset of collected ico**mpanion** data. We performed a non-parametric Kruskal–Wallis H test (or a one-way analysis of variance (ANOVA) on ranks), with MS type as independent variable and the variable of interest as dependent variable (mental feeling, physical feeling, prEDSS, SymptoMScreen composite, Neuro-QoL Fatigue, and Neuro-QoL Cognitive Function). We used a significance level of 0.05 and *p*-values of the separate models were corrected for multiple comparisons using false discovery rate (FDR) correction [30]. For the variables that showed significant group effects, post-hoc multiple comparisons tests were carried out for pairwise differences between the different MS types with Dunn–Sidák correction, using a significance level of 0.05.

The percentage of PwMS with a statistically meaningful change in their Neuro-QoL Fatigue and Cognitive Function scores was evaluated based on the conditional minimal detectable change, specifically developed for the Neuro-QoL short-forms [31]. A statistically meaningful change can be interpreted as a difference of more than one standard error (SE). It was estimated from the average of the SEs from a normative dataset for any given pair of scores multiplied by the z score for a 95% confidence interval, or $\left(\left[{SE}_{Score1}+{SE}_{Score2}\right]/2\right)\cdot 1.96\cdot \sqrt{2}$ [31]. Solely PwMS with more than one result for these PROs were included.

### 2.4. Monitoring Subclinical Disease Activity Using ico**brain ms**: Real-World Evidence

ico**brain ms** was launched in 2016 and has been adopted by more than 400 hospitals worldwide since then. In the context of standardizing MS care, in this manuscript, we evaluate how:

- ico**brain ms**’ lesion annotations bring the performance of non-specialized radiologists closer to that of experienced neuroradiologists (Section 2.4.1)
- the availability of ico**brain ms** reports in MS follow-up refine the assessment of subclinical disease activity (Section 2.4.2)
- how the use of quantitative AI based brain MRI reporting can improve the radiological workflows (Section 2.4.2)
- ico**brain ms** volumetric brain biomarkers bring insights into the brain patterns of MS types (Section 2.4.3)

#### 2.4.1. Study Synopsis-Reliability of Lesion Count with ico**brain ms**

ico**brain ms** lesion segmentations were compared with the assessment of two raters, one experienced radiologist and one assistant neurologist. The experiment consisted of marking and counting MS lesions on fluid-attenuated inversion recovery (FLAIR) and T1-weighted images acquired from 10 PwMS with a 3T MRI scanner (Achieva, Philips Medical Systems) at the University Hospital Brussels. Inclusion criteria were MS diagnosis according to McDonald Criteria 2010 and no MRI contraindication. For more details, see [27], from which a subset was used for this analysis. Two repeated acquisitions with patient repositioning were taken to assess test-retest reliability of the lesion count. The two raters independently assessed all images, which were presented in a shuffled order, first as original MRI scans, then with lesion annotations obtained by ico**brain ms**. In addition, the reporting time was recorded.

Intra-rater and inter-rater agreement of lesion counts was assessed. Of special interest was the question whether there is an improved agreement between the counts reported by the two raters after using ico**brain ms** segmentations, as opposed to the case when each rater counted lesions on the original images without ico**brain ms**.

#### 2.4.2. Study Synopsis-Detecting Subclinical Disease Activity in MRI Follow-Up with ico**brain ms**

In this study, we evaluated how the availability of ico**brain ms** reports might change the findings of radiological reading when assessing follow-up brain MRI scans. Longitudinal MRI acquisitions from 25 PwMS approximately 1 year apart were randomly selected (and limited to 25 because of feasibility) from different institutions that use ico**brain ms** in clinical practice, ensuring that these centers obtained informed consent from their PwMS to use fully anonymized MRI scans for research. The inclusion criteria were (1) being diagnosed as RRMS or SPMS, (2) having 2 pairs of scans separated at least one or more years apart acquired at the same scanner, (3) Having MRI acquired at the 1.5T or 3T in which there is a presence of high-resolution 3D-T1 and 2D or 3D FLAIR sequence, (4) having Expanded Disability Status Scale (EDSS) assessment at baseline and at follow-up, and (5) not having steroid treatment or relapse 30 days prior to the MRI scan. Each MRI dataset was presented in random order to an experienced neuroradiologist: once without and once with an automatically generated ico**brain ms** report. In the latter case, besides color-coded lesion and brain segmentation overlays, the expert also had access to the structured ico**brain ms** reports. The two radiological reporting scenarios (without and with volumetric software results) were compared in terms of effect on diagnostic findings and reporting time.

#### 2.4.3. Study Synopsis-Insights into the MS Brain Patterns Using ico**brain ms**

In this study, it was evaluated whether ico**brain ms** is able to reveal different brain or lesion volume patterns when comparing different MS clinical phenotypes. Multiple MR sessions from CIS (*n* = 12), RRMS (*n* = 30), PPMS (*n* = 17), and SPMS (*n* = 28) PwMS, with 3D T1w and 2D FLAIR images acquired on a 1.5T MR system (Sonata Siemens) at CERMEP in Lyon, were evaluated. EDSS was also available at each time point. For more details on the original dataset, see [32]. First, differences between MS groups were calculated in terms of longitudinal lesion evolution by location, where new and enlarging lesions were estimated between two time points at least 2 years apart for each patient. Secondly, we evaluated the known-groups validity or the sensitivity of ico**brain ms** to subclinical differences in brain volumetrics. This was done in the same way as for the ico**mpanion** data (see Section 2.3.1) using the Kruskal–Wallis H tests to look for a group effect, and pairwise post-hoc tests to look for differences between different MS type groups.

## 3. Results

### 3.1. Patient Perspective

#### 3.1.1. Patient Survey 1: Telemonitoring Tools for Monitoring Clinical Disease Activity

45 PwMS completed the survey, of which 80% (*n* = 36) were women. The average age of participants was 45.6 (SD = 11.5). Of the sample, 55.6% (*n* = 25) were RRMS, 15.6% (*n* = 7) were SPMS, 11.1% (*n* = 5) PPMS while 17.8% (*n* = 8) did not know their MS type or did not want to disclose it. About one third of PwMS were diagnosed in the last three years (31.1%, *n* = 14) or had a disease duration of 3 to 10 years (31.1%, *n* = 14), while 22.2% (*n* = 10) and 15.5% (*n* = 7) had been diagnosed, respectively, 10 to 20 years ago and longer than 20 years ago. The larger part of participants thought of themselves to be very digitally literate (33%, *n* = 15) or quite digitally literate (42.2%, *n* = 19) compared to neutral (22.2%, *n* = 10) and not quite digitally literate (2.2%, *n* = 1) while no PwMS indicated to be not very digitally literate.

**Figure 4:**
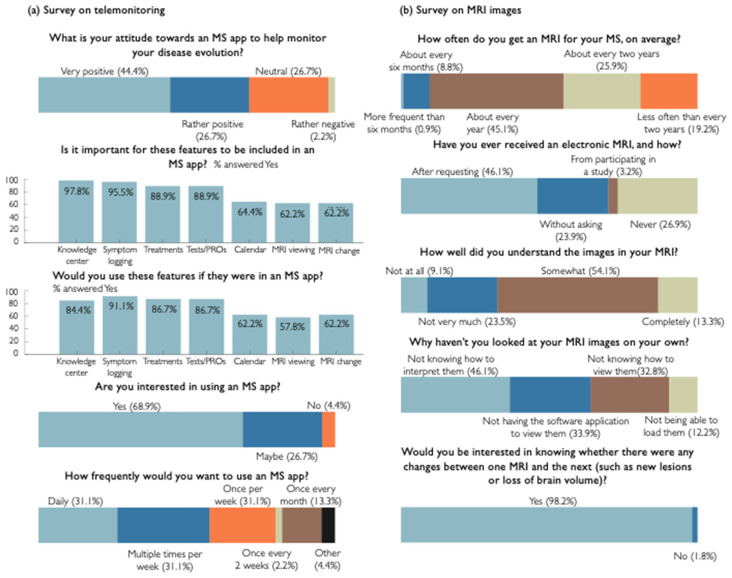
Visualization of answers for a selection of survey questions from the two surveys: (**a**) survey on telemonitoring tools for monitoring clinical disease activity, (**b**) survey on MR imaging for monitoring subclinical disease activity in collaboration with iConquerMS.

We asked for PwMS’ attitude about the use of an app to monitor the disease course, where only one person (2.2%) answered negatively (see Figure 4a). The most important features (see Figure 2 for an overview of the main functions) for this cohort were Knowledge center (97.8%, *n* = 44), Symptom logging (95.5%, *n* = 43), Treatment overview (88.9%, *n* = 40), and Test/PROs (88.9%, *n* = 40). These were also the features reported to be the most probable to be used by the PwMS. When asking whether PwMS had an intention to use the app, 68.9% (*n* = 31) answered yes, 26.7% (*n* = 12) answered maybe and 4.4% answered no (*n* = 2). When asking how frequently they would like to use the app, 26.7% (*n* = 12) answered daily, 31.1% (*n* = 14) multiple times per week, 22.2% once per week (*n* = 10), 2.2% (*n* = 1) once every two weeks and 13.3% (*n* = 6) once every month while 4.4% (*n* = 2) answered Other.

#### 3.1.2. Patient Survey 2: MR Imaging for Monitoring Subclinical Disease Activity

The survey was answered by 876 PwMS, predominantly located in the U.S. and Canada (91.4%). Of the participants, 80% (*n* = 699) were female. 2.8% of PwMS were aged 75 to 84 years, 19.4% 65 to 74 years, 34.1% 55 to 64 years, 27.2% 45 to 54 years, 11.7% 35 to 44 years, and 4.9% 34 years or younger.

The results of the survey showed that only 0.6% (*n* = 5) of PwMS have never had an MRI performed for the purpose of diagnosing or treating. Only 54.9% (*n* = 474) undergo an MRI scan every year or more frequently (see Figure 4b). Almost 27% (26.9%, *n* = 228) of PwMS have never received an electronic version of their MRI from their clinic or radiology lab. Of the PwMS that received an electronic version of their MRI, 79.9% (*n* = 560) got it on a CD-ROM, 15.6% (*n* = 109) through their clinic’s patient portal, 4.0% (*n* = 28) through a direct download into their computer or other device and 0.6% (*n* = 4) on a USB-drive.

Of PwMS that received an electronic version of their MRI, 70. 5% (*n* = 431) looked at their MR images on their own. Of those people, only 13.3% (*n* = 57) claimed to completely understand their MR images. 70.2% (*n* = 99) of the PwMS that had access to an electronic MRI but have not looked at it on their own, would like to do so. Of the reasons for not viewing the MR images, 46.1% (*n* = 83) of PwMS indicated to not know how to interpret the images, while 33.9% (*n* = 61) did not have a software application to view them, 32.8% (*n* = 59) did not know how to view the images, and 12.2% (*n* = 22) failed to load the images onto their computer or software program.

Respectively 98.2% (*n* = 836) and 94.7% (*n* = 767) of PwMS answered to be interested in knowing about changes between their MRIs and whether their MRI scan was performed according to clinical MS guidelines. Finally, 96.6% (*n* = 714) of PwMS indicated that they would be willing to share their MRI scans with researchers.

### 3.2. ico**mpanion** MS Patient App Validation

#### Sensitivity to Clinical Differences between MS Types

**Figure 5:**
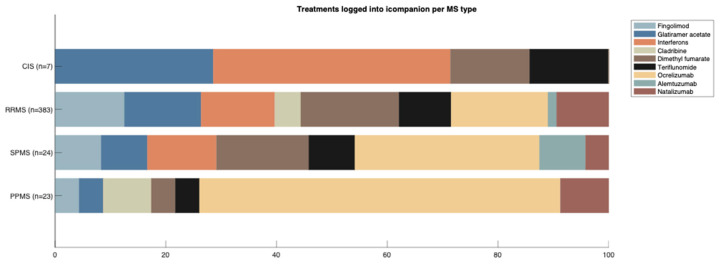
Treatments logged into ico**mpanion** by PwMS per MS type.

Summary statistics of ico**mpanion** users’ characteristics and entered data are presented in Table 1, including gender distribution, and average age and disease duration of the current user base. In Figure 5, the distribution of entered treatments per MS type is visualized, for PwMS on a DMT. For CIS, 28.6% were on glatiramer acetate, 42.9% on interferons, 14.3% on dimethyl fumarate, and 14.3% on teriflunomide. For people with RRMS, 12.5% indicated that they were on fingolimod, 13.8% on glatiramer acetate, 13.3% on interferons, 4.7% on cladribine, 17.8% on dimethyl fumarate, 9.4% on teriflunomide, 17.5% on ocrelizumab, 1.6% on alemtuzumab, and 9.4% on natalizumab. For people with SPMS, 8.3% indicated that they were on fingolimod, 8.3% on glatiramer acetate, 12.5% on interferons, 16.7% on dimethyl fumarate, 8.3% on teriflunomide, 33.3% on ocrelizumab, 8.3% on alemtuzumab and 4.2% on natalizumab. Finally, 4.4% of people with PPMS were on fingolimod, 4.3% on glatiramer acetate, 8.7% on cladribine, 4% on dimethyl fumarate or teriflunomide, 65.2% on ocrelizumab, and 9% on natalizumab.

**Figure 6:**
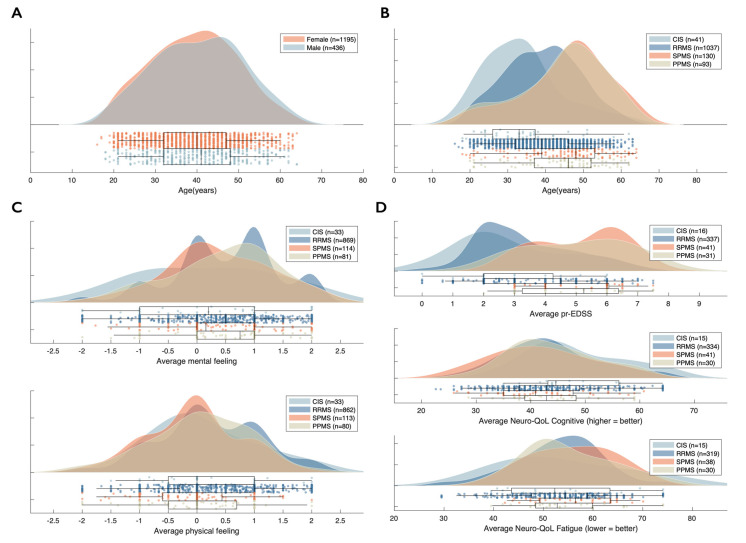
Summary statistics of ico**mpanion** users: (**A**) distribution of age based on sex, (**B**) distribution of age based on MS type, (**C**) distribution of average mental and physical feeling based on MS type, (**D**) distribution of average Neuro-QoL Cognitive and Fatigue score based on MS type.

In context of the validation of ico**mpanion** through known-groups validity, we observed a group effect of MS type on physical feeling (*p* = 0.025) (see Table 1 and Figure 6C). We also observed a significant group effect of MS type on body function or prEDSS (*p* < 0.001 Figure 6D) and general symptom load or SymptoMScreen composite (*p* = 0.005) where progressive PwMS scored higher than people with CIS and RRMS (Table 1). For mental feeling (*p* = 0.193) and the Neuro-QoL Fatigue (*p* = 0.312), we observed no effect of MS type. For the latter, average scores for all MS types were worse than the average general US reference sample, but within the range of 1 SD (50 ± 10) [23]. The same was true for average scores on the Neuro-QoL Cognitive where lower scores indicate worse functioning, and where also no effect of MS type was observed (*p* = 0.193).

Post-hoc tests for the variables that showed significant effects of MS type were carried out, performing pairwise comparisons between the different MS types. For physical feeling, we observed a significantly higher or better physical feeling in people with RRMS compared to SPMS (*p* = 0.014). prEDSS scores for both PPMS and SPMS were each significantly higher than both people with RRMS and CIS (all *p* < 0.001). Average symptom load, or SymptoMScreen composite, was significantly higher in people with PPMS compared to RRMS (*p* = 0.022).

For the Neuro-QoL PROs, we were able to calculate whether two consecutive scores indicated a statistically meaningful change (based on conditional minimal detectable change, described in Section 2.3.1 and [31]). We observed such statistically meaningful change in 25.0% (*n* = 36) of PwMS with more than 1 logged Neuro-QoL Fatigue result score (*n* = 118) and 12.3% (*n* = 18) of PwMS with more than 1 logged Neuro-QoL Cognitive score (*n* = 121).

**Figure 7:**
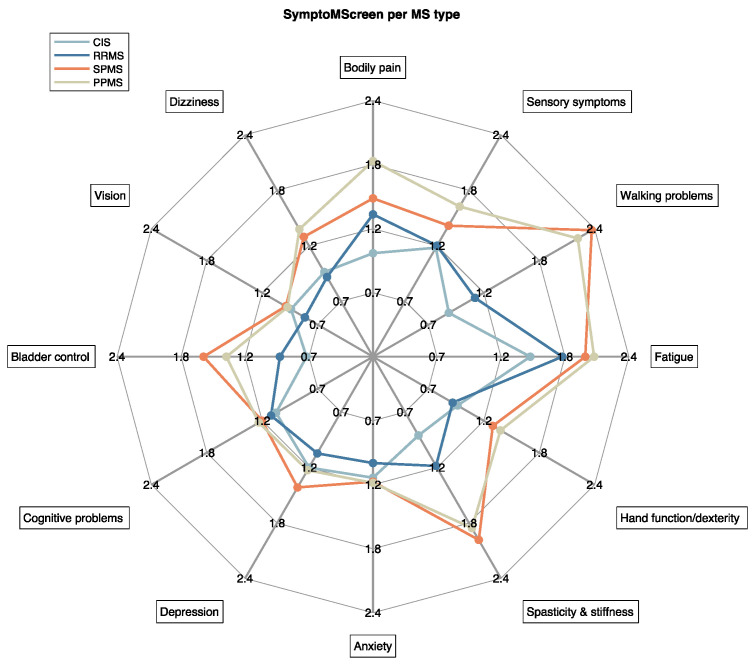
Average severity scored by ico**mpanion** users for all SymptoMScreen symptoms based on MS type. Severity is scored on a scale of 0–6.

The scores for the separate 12 symptoms included in the SymptoMScreen are shown in Figure 7. Visually, a clear distinction could be made between CIS and RRMS, and progressive MS types (SPMS, PPMS). Especially concerning Walking problems, SPMS (2.32) and PPMS (2.18) seem to score higher than CIS (0.86) and RRMS (1.13) on average, but also on Spasticity and stiffness (CIS: 0.89; RRMS: 1.20; SPMS: 1.96; PPMS: 1.84), Hand function and dexterity (CIS: 0.95; RRMS: 0.90; SPMS: 1.31; PPMS: 1.39) and Bladder control (CIS: 0.67; RRMS: 0.91; SPMS: 1.58; PPMS: 1.38).

### 3.3. ico**brain ms** Brain MRI Analysis Validation

#### 3.3.1. Reliability of Lesion Count with ico**brain ms**

**Figure 8:**
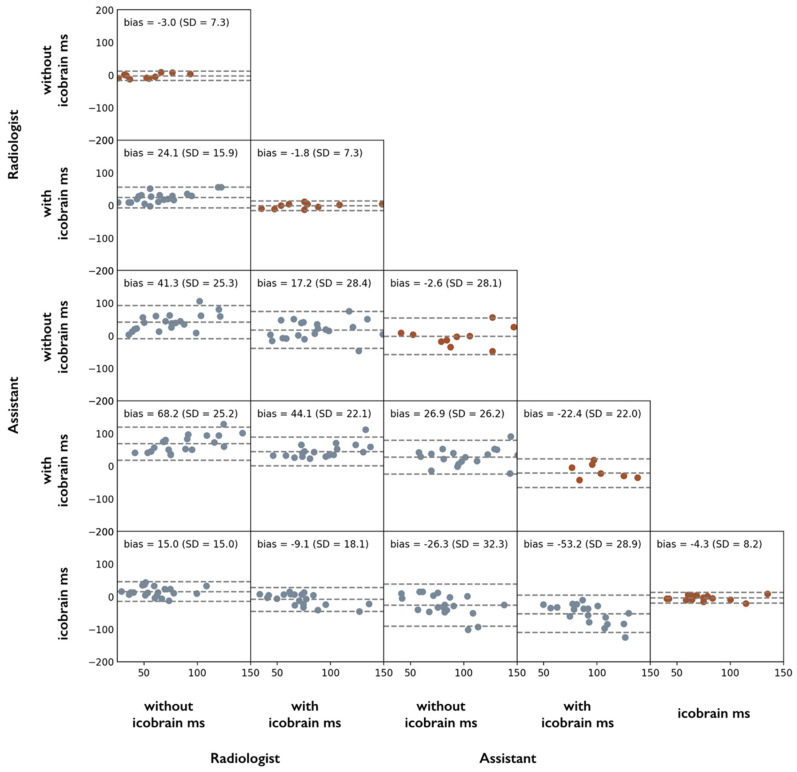
Bland–Altman plots of total FLAIR lesion counts on scan-rescan MRI data from 10 PwMS for 2 different raters (expert radiologist and assistant neurologist) and different scenarios per rater (without and with ico**brain ms**). The main diagonal depicts the intra-rater test-retest agreement over the 10 repeated MRI scans. Non-diagonal plots represent inter-rater or inter-scenario comparisons using the complete dataset of 20 MRI scans.

Intra-rater test-retest lesion count agreement on scan and rescan images was significantly improved for the assistant neurologist, from a standard deviation (SD) of the differences between test and retest lesion counts of 28.1 without ico**brain ms** to 22.0 with ico**brain ms** (improvement of 21.7%) but was constant for the experienced radiologist (SD = 7.3 in both scenarios). Larger changes were observed in the case of inter-rater agreement: without ico**brain ms** annotations, inter-rater lesion count agreement between experienced radiologist and assistant neurologist was significantly worse (SD = 20.8) than with ico**brain ms** (SD = 15.7), indicating an improvement of 32.5% by using ico**brain ms**. Figure 8 presents all intra- and inter-rater comparisons as Bland–Altman plots, annotated with bias and standard deviation of the lesion count differences, including the raters’ comparisons with the automated lesion count obtained from the ico**brain ms** lesion annotations. It can be observed that the assistant neurologist consistently overestimated the counts of ico**brain ms** and of the experienced radiologist. Similar trends were observed when repeating the analysis for T1 hypointensities (blackholes) and lesions per location, see [33].

The timing also differed significantly between the task of performing lesion count without (mean ± SD: 54.3 ± 11.8 min), or with ico**brain ms** (mean ± SD: 26.7 ± 19.8 min).

#### 3.3.2. Detecting Subclinical Disease Activity in MRI Follow-Up with ico**brain ms**

Radiological findings were compared between the scenario when the radiologist examined the raw MRI follow-up scans and the scenario when ico**brain ms** annotations and reports were also available. The PwMS were considered stable, slightly active, and active, as follows [34]:

- stable: if they had no new or enlarging lesions and had normal rate of brain atrophy compared to controls (within 0.2% from normal atrophy rate of sex- and age-matched healthy controls in the case of ico**brain ms** measurements);
- slightly active: if they had enlarging lesions or slightly abnormal atrophy rate compared to controls (further than 0.2% but within 0.4% from normal atrophy rate of sex- and age-matched healthy controls in the case of ico**brain ms** measurements);
- active: if they had new lesions or severe progression of brain atrophy (further than 0.4% from the normal atrophy rate of sex- and age-matched healthy controls in the case of ico**brain ms** measurements).

Conventional radiological reporting indicated 19 out of 25 stable PwMS (no lesion activity, no apparent atrophy) and 6 active PwMS (new lesion formation or lesion enlargement). The radiological findings with access to ico**brain ms** indicated 7 out of 25 PwMS as stable (normal atrophy, no lesion activity), 7 PwMS with slight disease activity (slightly abnormal atrophy rate and/or enlarging lesions), and 11 active PwMS (5 with new lesions, 10 with abnormal atrophy rate for their age). All stable PwMS identified by ico**brain ms** were also deemed stable by conventional radiological reading. All active PwMS identified by conventional reading were also identified as active or slightly active when using ico**brain ms**. However, the automatic brain MRI measurements indicated several other PwMS as (slightly) active, even if these were part of the stable group according to conventional radiological reading. As such, the percentage of PwMS deemed as having (slight) disease activity or progression grew from 24% in conventional reading to 76% (44% active, 32% slightly active, according to the definitions above) with the ico**brain ms** assisted reading.

With respect to timing, radiological reporting took on average 7 min 28 s (SD: 3 min 6 s) without ico**brain ms** and 5 min 49 s (SD: 2 min 15 s) with ico**brain ms**. In other words, computer-aided radiological reporting with ico**brain ms** was faster than conventional reporting, with approximately 8 conventional reports per hour versus 13 computer-aided reports per hour, which is an improvement by about 40%.

#### 3.3.3. Insights into the MS Brain Patterns: Sensitivity to Subclinical Differences between MS Types

**Figure 9:**
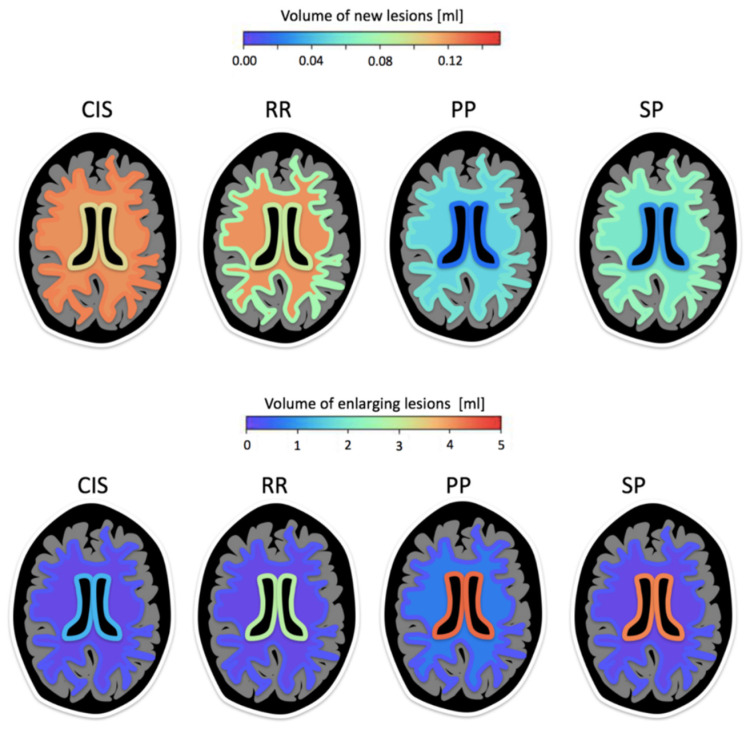
Location prevalence and average volumes for new and enlarging lesions in subjects with CIS, RRMS, PPMS, and SPMS. The schematic representation shows three colored layers, where colors represent new or enlarging lesion volumes in the given scales (mL). For each brain slice, the innermost contour represents periventricular lesions; the outermost contour represents juxtacortical lesions; and middle region represents deep white matter lesions.

In a two-year MRI follow-up study, new and enlarging lesions assessed with ico**brain ms** were evaluated in different MS subtypes of CIS (*n* = 12), RRMS (*n* = 30), PPMS (*n* = 17), and SPMS (*n* = 28) PwMS, with average age at baseline 31.8, 33.2, 39.5, and 41.1 years and average disease duration 2.9, 8.3, 7.5, and 14.9, respectively [35]. The largest volume of new lesion formation (i.e., lesions not touching any older lesion) was observed in CIS, with approximately 0.1ml new lesion volume over 2-year follow-up, without a preferred location (juxtacortical, periventricular, deep white matter). Further, it was also observed that people with RRMS exhibited more deep white matter (WM) lesions (either new or pre-existing) in comparison to other MS types. PPMS and SPMS had virtually no new periventricular lesions, but a significant amount of enlargement in that region, consistent with a longer disease duration. Figure 9 illustrates the location-dependent evolution patterns for new and enlarging lesions obtained with ico**brain ms** in the 4 MS clinical phenotypes.

**Figure 10:**
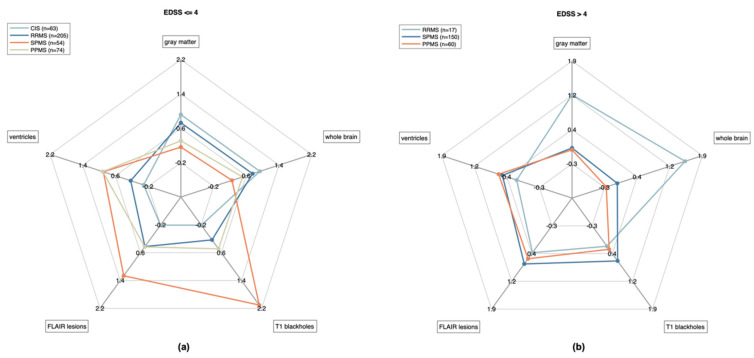
Volumetric patterns observed with ico**brain ms** are illustrated for (**a**) EDSS lower than or equal to 4 and (**b**) greater than 4 (right). For each considered volume (whole brain, gray matter, lateral ventricles, FLAIR lesions, T1 blackholes), the represented radius goes from low volume in the center to high volume on the exterior (each volume being re-scaled based on 1st and 3rd quartiles in the four groups combined). Solid lines link the median volumes per group.

When examining brain and lesion volumes simultaneously (whole brain volume, gray matter volume, lateral ventricles volume, total FLAIR lesion volume, and T1 blackholes volume), very distinct group patterns were observed for the MRIs corresponding to all time points for which EDSS was lower than or equal to 4 (Figure 10a), with all volumes significantly different between groups according to a non-parametric Kruskal–Wallis H test with MS type as independent variable (*p* < 0.001) (Table 2). The CIS and RRMS groups showed significantly higher whole brain and gray matter volumes and lower ventricular volumes compared to the PPMS and SPMS groups (Table 2). Highest volumes of FLAIR hyperintensities and T1 blackholes were evident in SPMS. At higher EDSS (greater than 4), the patterns corresponding to PPMS and SPMS groups were almost indistinguishable, with no significant volume differences observed for whole brain, gray matter, lateral ventricles and T1 blackholes (Table 2). In contrast, the RRMS group with EDSS > 4 showed higher whole brain volume and lower ventricles volume compared to the progressive groups.

## 4. Discussion

Digital solutions have the potential to assist clinicians to further standardize MS clinical decision making, to allow for an early detection of disease activity and inform therapeutic decisions. As these solutions are now available with the necessary regulatory clearances and hospital integrations, they can be used in a routine clinical setting.

In this paper, we present the initial real-world evidence results of such a novel regulatory cleared and workflow-integrated MS care path solution. It was demonstrated that the ico**mpanion** mHealth application is a response to clear patient needs and that it is a sensitive tool to capture clinically relevant information about MS symptoms and patient wellbeing, as well as significant longitudinal changes in cognition and fatigue over time. In addition, it was shown that ico**brain ms**’ MRI volumetric brain reports save radiologists 40% time while also detecting subclinical MRI activity with a significantly higher sensitivity.

### 4.1. Patient Perspective

In order to gain insight into the PwMS’ perspective on digital telemonitoring solutions, we carried out a survey which was answered by 45 PwMS of which the larger part (75.2%) indicated to see themselves as digitally literate. Only one patient (2.2%) indicated to have a negative attitude towards using an app to monitor their disease course, which is in line with previous reports about positive attitudes of PwMS [13].

Patients reported the most important features to be a knowledge center, symptom logging, tests/PROs and treatment overviews (88–98%), but more than 60% also found having an appointment calendar, viewing their own MRI scans, and viewing the evolution of their MRI scan important. The features that PwMS thought they would actually use were symptom logging, performing tests/PROs, treatment overviews and a knowledge center were found to be most popular (84–91%). This is in line with recent research indicating a patient demand for medication schedules and reminders [13]. This study also indicated a strong interest for visit overviews, which has been implemented into ico**mpanion**’s calendar and visit preparation feature recently. What differentiates ico**mpanion** from other MS apps available is that ico**mpanion** integrates all the features mentioned above and, at the same time, is a CE-marked and FDA-cleared medical device.

From PwMS in our cohort, 68.9% indicated to intend to start using an MS app like ico**mpanion**, and 80.1% intended to start using it daily or weekly which is in line with a previous study [36]. While our survey suggests that PwMS are interested in telemonitoring apps and their features, and actually using them, it must be noted that the sample size of this survey was relatively small and potentially biased due to the relatively small number of non-digitally literate PwMS.

A second survey was carried out to gain insight into PwMS’ perspective on MRI scans in collaboration with iConquerMS. The survey investigated PwMS’ experiences with MRI scans as well as their knowledge and viewing behavior and was answered by a total of 876 PwMS. Responses indicated that about 45% of PwMS did not have a yearly brain MRI scan, as advised by the MAGNIMS-CMSC-NAIMS recommendations [11]. Of the PwMS who received an electronic version of their MRI, 70.5% looked at their images on their own, but only 13.3% of PwMS reported to completely understand these MR images, in line with previous studies [37]. Considering the key role that MRI plays in clinical decisions in MS care, and the positive outcomes related to an increased patient involvement in clinical decisions [38,39], it is important to include an MRI-focused knowledge center and MRI viewer in medical apps for MS.

Relevant to the latter, our survey showed that many technological limitations prevented PwMS from looking at their MRI scans on their own. 33.9% of PwMS reported not having a software application to view the images, 32.8% not knowing how to view the images, and 12.2% failing to load the images onto their computer or software program. 94.7% of PwMS indicated to be interested in knowing about whether their MRI scan was performed according to clinical guidelines. This is relevant to PwMS, providers and payers, as it has been demonstrated that less than 10% of the MRI scans for PwMS were acquired according to the local guidelines [40]. Finally, almost all PwMS (98.2%) indicated their interest in knowing about changes between their MRIs. This information is provided to PwMS’ care teams via ico**brain ms** in the MS care platform.

### 4.2. icompanion MS Patient App Validation

In a first exploratory analysis aimed at investigating the validity and clinical relevance of the real-world collected ico**mpanion** data, we assessed the sensitivity of ico**mpanion** PROs (mental and physical feeling, prEDSS, SymptoMScreen composite, Neuro-QoL Fatigue and Neuro-QoL Cognition) to clinical differences between MS types, so-called known-groups validity. A significant effect of MS type on physical feeling, prEDSS and SymptoMScreen composite was observed. We found physical feeling to be significantly worse in people with SPMS compared to RRMS, while prEDSS scores for both RRMS and CIS showed to be significantly lower than both PPMS and SPMS. These results are in line with previous studies that described a significant effect of MS type on EDSS [38,39,41]. Average symptom load or SymptoMScreen composite was found to be significantly higher in people with PPMS compared to RRMS, which is in line with a previous study [39] that found that scores for symptoms associated with spinal cord abnormalities were significantly higher for SPMS and PPMS than for RRMS. These symptoms were included in the SymptoMScreen as Spasticity and stiffness, Sensory symptoms, and Bladder control, and consequently also in the SymptoMScreen composite [21]. While these findings are expected, and in line with the literature described above, they indicate that the prEDSS [22] and SymptoMScreen [21] PROs included in the ico**mpanion** mobile app are able to pick up important clinical differences between MS types.

In addition, we observed statistically meaningful changes, based on conditional minimal detectable change [31], for the Neuro-QoL (V1.0) Fatigue in 25.0% of PwMS with more than one datapoint, and in 12.3% of PwMS with more than one datapoint for the Neuro-QoL (V2.0) Cognitive. This is the first time that this measure, aimed at providing a clinically useful way of interpreting individual change in the Neuro-QoL short-forms, is employed in MS, and this suggests that ico**mpanion** is able to pick up statistically meaningful and consequently clinically relevant changes in cognition and fatigue in PwMS. In the HCP portal, HCPs can easily evaluate whether changes in the data entered by linked PwMS for these PROs are statistically meaningful based on this measure. This provides them with an indication of changes in clinical symptoms that are large enough to help motivate treatment changes [31].

In summary, we provide real-world data obtained by a medical device app which are in line with other published studies and provide initial evidence that it is feasible to obtain reliable real-world data which can potentially be used for clinical decision making. Further in-depth analyses will be needed on how mHealth app telemonitoring data can help PwMS, inform clinicians, and impact clinical decision making and outcomes.

### 4.3. icobrain ms Brain MRI Analysis Validation

The use of follow-up brain MRI scans to detect disease activity in PwMS is recommended by all international guidelines. Typically, changes in terms of new and enlarging lesions and brain volume compared to the previous brain scan are evaluated visually. However, especially because many lesions can be present in an MS patient’s brain and subtle but significant brain atrophy is almost impossible to visually assess, it is known that visual MRI reading is prone to inter-rater variability and potential discrepancies [12]. This was confirmed by the results reported in this paper, which demonstrate a significant inter-rater lesion count difference, which can in part be explained by a subjective rater’s preference for merging certain nearby lesions into one connected lesion, or for indicating separate nearby lesion foci as distinct lesions. It should be mentioned here that in the presented experiment the raters were asked to provide the best possible lesion count as possible, not to perform a brain MRI reading they would do in a clinical setting. Given the time pressure and distractions in a clinical context, it can be expected that the variability in detecting (new) lesions can be even higher. In this study, it was demonstrated that ico**brain ms** has an excellent test-retest lesion count agreement, and that the expert raters improved their test-retest lesion count agreement when the software annotations were made available. Such results are in line with previous studies that used various other assistive research software approaches [42,43,44,45] although we must note that one limitation of this analysis was the small number of raters.

As detecting brain MRI based disease activity is an essential part of the current MS treatment guidelines, it is important to assess to what extent AI augmented radiological reading can impact clinical decision making. In this context, it was demonstrated that the use of ico**brain ms** together with the radiological reading detected a significantly higher number of PwMS with disease activity when compared to the visual radiological assessment alone. This is in line with the results reported by [46], where the proportion of PwMS who were found as having evidence of disease activity/progression grew from around 35% based on clinical criteria alone to around 54% based on conventional radiological reading (with lesion activity and/or visually estimated brain atrophy), to 61% and 80% when employing radiological reading assisted by ico**brain ms** (only lesion activity, and lesion activity and estimated annual atrophy thresholded at 0.4%, respectively). In addition, and as crucial to implement new technologies in the clinical setting, it was observed that the radiological reading workflow was improved by 40%, which is significant given the increasing time pressure on radiologists [47].

Finally, known-groups validity was also demonstrated for ico**brain ms** (Table 2) as significant group differences were observed between MS phenotypes for the different ico**brain ms** volumetric measures (Figure 10). These findings, albeit based on limited sample sizes, indicated that lesion evolution and brain volumetry, as well as cognitive performance and symptoms, have distinct patterns in the relapsing and progressive types/phases of MS, but that the patterns seem to become more indistinguishable once the disease is more advanced. Indeed, there is more heterogeneity in brain atrophy and lesion burden patterns among different MS groups (relapsing-remitting, primary progressive, secondary progressive) at low EDSS, and, conversely, brain atrophy and lesion burden patterns converge to a common pattern when EDSS gets higher (Figure 10). The RRMS group is clearly distinct from the progressive forms of MS. This divergence, followed by unification of clinical and subclinical findings, is in line with the unifying concept of MS [48]. This highlights the importance of not allowing the disease to progress beyond a certain stage, by addressing the earliest signs of disease activity before irreversible damage sets in.

The results from these analyses demonstrate that it is feasible to implement brain MRI AI solutions in a clinical routine setting and that they can improve the radiological workflow. In addition, it is shown that the ico**brain ms** software, as an assistive tool for radiological reading, decreases the intra- and inter-rater radiological reading variability. Finally, it was demonstrated that ico**brain ms** results can help differentiate between MS subtypes, in line with the literature, and that they allow for a significantly higher detection rate of MS disease activity.

In further research, we aim to provide the combined ico**mpanion** and ico**brain ms** results to clinicians to evaluate the potential impact of these technologies on clinical decision making and standardization of care.

## 5. Conclusions

Given the heterogeneity of the disease, the increasing number of available treatment options, and the long-term outcome effects of early clinical decisions in chronic disorders such as MS, there is a clear need to move towards more personalized decision making in MS. Hence, MS care pathways need to become more data-driven and standardized. In this paper, real-world evidence on how new digital/AI technologies can impact MS patient care was presented, and the feasibility of linking different digital tools into one overarching MS care pathway was demonstrated.

## Figures and Tables

**Table 1:** Descriptive statistics for the complete dataset of app users that indicated to know their MS type (82.8%, *n* = 1301). Except for gender, all variables are described as [average (standard deviation, *n*)]. Group effect column provides the results of the Kruskal–Wallis analyses to look for an effect of MS type on these variables. *p*-values have been corrected using FDR correction.

	CIS 3.1% *n* = 42	RRMS 78.4% *n* = 1061	SPMS 10.9% *n* = 147	PPMS 7.6% *n* = 103	Group Effect
Gender (female)	76.2%	75.7%	61.2%	63.1%	
Age	33.9 (10.2, 41)	39.7 (10.4, 1058)	47.7 (12.6, 144)	45.8 (12.6, 101)	
Disease duration	5.24 (6.87, 42)	7.98 (6.84, 1061)	16.5 (9.21, 147)	7.38 (6.25, 103)	
Mental feeling	0.17 (1.17, 33)	0.49 (0.91, 869)	0.37 (0.89, 114)	0.47 (0.91, 81)	H(3) = 5.15 *p* = 0.193
Physical feeling	0.12 (0.89, 33)	0.25 (0.88, 862)	−0.05 (0.81, 113)	0.06 (0.85, 80)	H(3) = 10.85 *p* = 0.025
prEDSS	2.87 (1.77, 16)	3.35 (1.57, 336)	5.13 (1.38, 41)	5.02 (1.62, 31)	H(3) = 64.47 *p* < 0.001
Sympto-MScreen composite	10.5 (13.2, 40)	12.8 (13.5, 1024)	17.0 (15.5, 140)	17.9 (15.9, 101)	H(3) = 15.40 *p* = 0.005
Neuro-QoL Fatigue	54.0 (11.91, 15)	55.1 (8.75, 319)	56.0 (7.74, 38)	55.1 (8.95, 30)	H(3) = 0.31 *p* = 0.312
Neuro-QoL Cognitive	48.5 (8.95, 15)	43.7 (8.87, 334)	42.0 (9.09, 41)	44.2 (7.97, 30)	H(3) = 5.22 *p* = 0.193

**Table 2:** Results from statistical analysis of differences in ico**brain ms** volumetrics between MS types. The group effect rows provide the results of the Kruskal–Wallis analyses looking for an effect of MS type on these variables. Other rows indicate the group differences as observed via post-hoc pairwise tests. *p*-values have been corrected for multiple comparisons.

	Whole Brain	Gray Matter	Lateral Ventricles	FLAIR Lesions	T1 Blackholes
**EDSS ≤ 4**					
Group effect	*p* < 0.001	*p* < 0.001	*p* < 0.001	*p* < 0.001	*p* < 0.001
CIS vs. RRMS	*p* = 0.008	*p* = 0.258	*p* = 0.029	*p* < 0.001	*p* < 0.001
CIS vs. SPMS	*p* < 0.001	*p* < 0.001	*p* < 0.001	*p* < 0.001	*p* < 0.001
CIS vs. PPMS	*p* < 0.001	*p* < 0.001	*p* < 0.001	*p* < 0.001	*p* < 0.001
RRMS vs. SPMS	*p* < 0.001	*p* < 0.001	*p* < 0.001	*p* < 0.001	*p* < 0.001
RRMS vs. PPMS	*p* < 0.001	*p* < 0.001	*p* < 0.001	*p* < 0.001	*p* = 0.173
SPMS vs. PPMS	*p* = 0.935	*p* = 1.000	*p* = 1.000	*p* = 0.902	*p* < 0.001
**EDSS > 4**					
Group effect	*p* = 0.013	*p* = 0.072	*p* = 0.014	*p* = 0.013	*p* = 0.054
RRMS vs. SPMS	*p* = 0.005	*p* = 0.073	*p* = 0.006	*p* = 0.034	*p* = 0.101
RRMS vs. PPMS	*p* = 0.007	*p* = 0.094	*p* = 0.027	*p* = 0.716	*p* = 0.662
SPMS vs. PPMS	*p* = 0.992	*p* = 1.000	*p* = 0.955	*p* = 0.033	*p* = 0.251

## Data Availability

The data presented in this study are not publicly available due to PwMS’ privacy rights.

## Appendix A

Note that these questions are part of a broader questionnaire that surveyed PwMS’ expectations of an MS app.

General

- What is your sex?
  ○ *Male*
  ○ *Female*
  ○ *Other*
- What is your age?
- When were you diagnosed with MS?
  ○ Less than 6 months ago
  ○ 6 months–1 year ago
  ○ 1–3 years ago
  ○ 3–5 years ago
  ○ 5–10 years ago
  ○ 10–15 years ago
  ○ 15–20 years ago
  ○ Over 20 years ago
  ○ I don’t know
  ○ I prefer not to share this information
- Which type of MS do you have?
  ○ Primary Progressive MS or PPMS
  ○ Secondary Progressive MS or SPMS
  ○ Relapsing Remitting MS or RRMS
  ○ I don’t know
  ○ I prefer not to share this information

Attitude towards MS application

- What is your attitude towards an MS app to help monitor your disease evolution?
  ○ *Very negative*
  ○ *Rather negative*
  ○ *Neutral*
  ○ *Rather positive*
  ○ *Very positive*

Features

- Is it important that a knowledge center is included in an MS app?
  ○ *Yes*
    ⯀ Would you use this feature?
      - *Yes*
      - *No*
  ○ *No*
- Is it important that symptom logging is included in an MS app?
  ○ *Yes*
    ⯀ Would you use this feature?
      - *Yes*
      - *No*
  ○ *No*
- Is it important that an overview and analysis of your MRI scans is included in an MS app?
  ○ *Yes*
    ⯀ Would you use this feature?
      - *Yes*
      - *No*
  ○ *No*
- Is it important that the evolution of your MRI scans is included in an MS app?
  ○ *Yes*
    ⯀ Would you use this feature?
      - *Yes*
      - *No*
  ○ *No*
- Is it important that tests are included in an MS app?
  ○ *Yes*
    ⯀ Would you use this feature?
      - *Yes*
      - *No*
  ○ *No*
- Is it important that a calendar to plan your doctor’s visits is included in an MS app?
  ○ *Yes*
    ⯀ Would you use this feature?
      - *Yes*
      - *No*
  ○ *No*
- Are you interested in an MS app?
  ○ *Yes*
  ○ *Maybe*
  ○ *No*
- How frequently would you use an MS app?
  ○ *Daily*
  ○ *Multiple times per week*
  ○ *Once per week*
  ○ *Once every two weeks*
  ○ *Once per month*
  ○ *Other*

## Appendix B

In case of fixed possible answers, see italic.

- Have you ever had an MRI performed for the purpose of diagnosing or treating your MS?
- How often do you get an MRI for your MS, on average? Please disregard any recent delays in getting an MRI that you may have experienced due to the COVID-19 pandemic.
- When did you last get an MRI of your brain?
- When did you last get an MRI of your neck or spinal column?
- Have you ever received or been given access to an electronic copy of your MRI? Check all that apply.
  ○ *Yes, my clinic or radiology lab gave it to me without my requesting it*
  ○ *Yes, my clinic or radiology lab gave it to me after I requested it*
  ○ *Yes, I got one from participating in a research study*
  ○ *No*
  ○ *Not sure/prefer not to say*
- Have you ever received or been given access to an electronic copy of your MRI? Check all that apply.
  ○ *CD/disc*
  ○ *USB drive/thumb drive*
  ○ *Direct download into my computer or other device*
  ○ *Access through my clinic’s patient portal*
  ○ *Other—Write In*
  ○ *Not sure/prefer not to say*
- Have you ever looked at your own MRI images on your own, without your healthcare provider present?
  ○ [in case of Yes] Did you find it helpful in any way to view your MRI images on your own? If so, how?
  ○ [in case of Yes] Did you find it unhelpful in any way to view your MRI images on your own? If so, how?
  ○ [in case of Yes] How well did you understand the images on your MRI?
  ○ [in case of No] Why haven’t you looked at your MRI images on your own? Check all that apply.
    ⯀ *I wasn’t sure how to view the images*
    ⯀ *I didn’t have a software program or application for viewing the files*
    ⯀ *I couldn’t load the images onto my computer or another device where I could view them*
    ⯀ *I didn’t know how to interpret the images*
    ⯀ *I wasn’t interested in viewing my MRI images on my own*
    ⯀ *Not sure/prefer not to say:*
    ⯀ *Other—Write In*
  ○ [in case of No] Would you like to view your MRI images on your own? Why or why not?
- Would you be interested in having your own copy of your MRI images to view?Why or why not?
- If you have or had access to your own MRIs, would you be interested in knowing whether the MRI was performed according to the current clinical guidelines for MS? Why or why not?
- If you have or had access to your own MRIs, would you be interested in knowing whether there were any changes between one MRI and the next (such as new lesions or loss of brain volume)? Why or why not?
- If you have or had access to your own MRIs, would you be willing to share your electronic MRI with a researcher, if asked? Why or why not?
- If you have or had access to your own MRIs, would you be willing to share them with iConquerMS, if asked, and allow iConquerMS to share them with researchers? Why or why not?

## References

[1] Coetzee T., Thompson A.J. (2020). Atlas of MS 2020: Informing Global Policy Change. Mult. Scler..

[2] National MS Society (Medications). https://www.nationalmssociety.org/Treating-MS/Medications.

[3] Hult K. (2017). Measuring the Potential Health Impact of Personalized Medicine: Evidence from MS Treatments.

[4] Sá M.J., de Sá J., Sousa L. (2014). Relapsing–Remitting Multiple Sclerosis: Patterns of Response to Disease-Modifying Therapies and Associated Factors: A National Survey. Neurol. Ther..

[5] Daugherty K.K., Butler J.S., Mattingly M., Ryan M. (2005). Factors Leading Patients to Discontinue Multiple Sclerosis Therapies. J. Am. Pharm. Assoc..

[6] Giovannoni G., Butzkueven H., Dhib-Jalbut S., Hobart J., Kobelt G., Pepper G., Sormani M.P., Thalheim C., Traboulsee A., Vollmer T. (2016). Brain Health: Time Matters in Multiple Sclerosis. Mult. Scler. Relat. Disord..

[7] Duddy M., Lee M., Pearson O., Nikfekr E., Chaudhuri A., Percival F., Roberts M., Whitlock C. (2014). The UK Patient Experience of Relapse in Multiple Sclerosis Treated with First Disease Modifying Therapies. Mult. Scler. Relat. Disord..

[8] Amato M.P., Ponziani G., Siracusa G., Sorbi S. (2001). Cognitive Dysfunction in Early-Onset Multiple Sclerosis: A Reappraisal after 10 Years. Arch. Neurol..

[9] Magnano I., Aiello I., Piras M.R. (2006). Cognitive Impairment and Neurophysiological Correlates in MS. J. Neurol. Sci..

[10] Kürtüncü M., Tuncer A., Uygunoğlu U., Çalişkan Z., Paksoy A.K., Efendı H., Kocaman A.S., Özcan C., Terzı M., Turan Ö.F. (2017). Differences Between General Neurologists and Multiple Sclerosis Specialists in the Management of Multiple Sclerosis Patients: A National Survey. Noro psikiyatri arsivi.

[11] Wattjes M.P., Ciccarelli O., Reich D.S., Banwell B., de Stefano N., Enzinger C., Fazekas F., Filippi M., Frederiksen J., Gasperini C. (2021). 2021 MAGNIMS–CMSC–NAIMS Consensus Recommendations on the Use of MRI in Patients with Multiple Sclerosis. Lancet Neurol..

[12] Rosenkrantz A.B., Duszak R., Babb J.S., Glover M., Kang S.K. (2018). Discrepancy Rates and Clinical Impact of Imaging Secondary Interpretations: A Systematic Review and Meta-Analysis. J. Am. Coll. Radiol..

[13] Haase R., Voigt I., Scholz M., Schlieter H., Benedict M., Susky M., Dillenseger A., Ziemssen T. (2021). Profiles of eHealth Adoption in Persons with Multiple Sclerosis and Their Caregivers. Brain Sciences.

[14] Ziemssen T., Thomas K. (2017). Treatment Optimization in Multiple Sclerosis: How Do We Apply Emerging Evidence?. Expert Rev. Clin. Immunol..

[15] D’Amico E., Haase R., Ziemssen T. (2019). Review: Patient-Reported Outcomes in Multiple Sclerosis Care. Mult. Scler. Relat. Disord..

[16] Celius E.G., Thompson H., Pontaga M., Langdon D., Laroni A., Potra S., Bharadia T., Yeandle D., Shanahan J., van Galen P. (2021). Disease Progression in Multiple Sclerosis: A Literature Review Exploring Patient Perspectives. Patient Prefer. Adherence.

[17] Hamann J., Neuner B., Kasper J., Vodermaier A., Loh A., Deinzer A., Heesen C., Kissling W., Busch R., Schmieder R. (2007). Participation Preferences of Patients with Acute and Chronic Conditions. Health Expect..

[18] van Leeuwen K.G., Schalekamp S., Mjcm R., van Ginneken B., de Rooij M. (2021). Artificial Intelligence in Radiology: 100 Commercially Available Products and Their Scientific Evidence. Eur. Radiol..

[19] Medina L.D., Torres S., Alvarez E., Valdez B., Nair K.V. (2019). Patient-Reported Outcomes in Multiple Sclerosis: Validation of the Quality of Life in Neurological Disorders (Neuro-QoL^TM^) Short Forms. Mult Scler J Exp Transl Clin.

[20] Miller D.M., Bethoux F., Victorson D., Nowinski C.J., Buono S., Lai J.-S., Wortman K., Burns J.L., Moy C., Cella D. (2016). Validating Neuro-QoL Short Forms and Targeted Scales with People Who Have Multiple Sclerosis. Mult. Scler..

[21] Green R., Kalina J., Ford R., Pandey K., Kister I. (2017). SymptoMScreen: A Tool for Rapid Assessment of Symptom Severity in MS Across Multiple Domains. Appl. Neuropsychol. Adult.

[22] Leddy S., Hadavi S., McCarren A., Giovannoni G., Dobson R. (2013). Validating a Novel Web-Based Method to Capture Disease Progression Outcomes in Multiple Sclerosis. J. Neurol..

[23] Cella D., Lai J.-S., Nowinski C.J., Victorson D., Peterman A., Miller D., Bethoux F., Heinemann A., Rubin S., Cavazos J.E. (2012). Neuro-QOL: Brief Measures of Health-Related Quality of Life for Clinical Research in Neurology. Neurology.

[24] Jain S., Sima D.M., Ribbens A., Cambron M., Maertens A., Van Hecke W., De Mey J., Barkhof F., Steenwijk M.D., Daams M. (2015). Automatic Segmentation and Volumetry of Multiple Sclerosis Brain Lesions from MR Images. NeuroImage Clin..

[25] Rakić M., Vercruyssen S., Van Eyndhoven S., de la Rosa E., Jain S., Van Huffel S., Maes F., Smeets D., Sima D.M. (2021). Icobrain Ms 5.1: Combining Unsupervised and Supervised Approaches for Improving the Detection of Multiple Sclerosis Lesions. Neuroimage Clin.

[26] Smeets D., Ribbens A., Sima D.M., Cambron M., Horakova D., Jain S., Maertens A., Van Vlierberghe E., Terzopoulos V., Van Binst A.-M. (2016). Reliable Measurements of Brain Atrophy in Individual Patients with Multiple Sclerosis. Brain Behav..

[27] Lysandropoulos A.P., Absil J., Metens T., Mavroudakis N., Guisset F., Van Vlierberghe E., Smeets D., David P., Maertens A., Van Hecke W. (2016). Quantifying Brain Volumes for Multiple Sclerosis Patients Follow-up in Clinical Practice—Comparison of 1.5 and 3 Tesla Magnetic Resonance Imaging. Brain Behav..

[28] Beadnall H.N., Wang C., Van Hecke W., Ribbens A., Billiet T., Barnett M.H. (2019). Comparing Longitudinal Brain Atrophy Measurement Techniques in a Real-World Multiple Sclerosis Clinical Practice Cohort: Towards Clinical Integration?. Ther. Adv. Neurol. Disord..

[29] Costers L., Schmidt H., Loud S., McBurney R., Gwynne D., Van Vlierberghe E., Descamps A., Smeets D., Van Hecke W. (2021). MRI in MS Survey—Insights into Access, Understanding and Interest by People with MS. Mult. Scler. J..

[30] Benjamini Y., Hochberg Y. (1995). Controlling the False Discovery Rate: A Practical and Powerful Approach to Multiple Testing. J. R. Stat. Soc..

[31] Kozlowski A.J., Cella D., Nitsch K.P., Heinemann A.W. (2016). Evaluating Individual Change with the Quality of Life in Neurological Disorders (Neuro-QoL) Short Forms. Arch. Phys. Med. Rehabil..

[32] Ion-Mărgineanu A., Kocevar G., Stamile C., Sima D.M., Durand-Dubief F., Van Huffel S., Sappey-Marinier D. (2017). Machine Learning Approach for Classifying Multiple Sclerosis Courses by Combining Clinical Data with Lesion Loads and Magnetic Resonance Metabolic Features. Front. Neurosci..

[33] Sima D.M., Podevyn F., Torcida N., Wilms G., Lysandropoulos A., Van Hecke W., Smeets D. Impact of MSmetrix Automatic Lesion Segmentation on the Visual Count of Multiple Sclerosis Lesions. Proceedings of the 2018 European Congress of Radiology.

[34] Sima D.M., Wilms G., Vyvere T.V., Van Hecke W., Smeets D. On the Use of Icobrain’s Prepopulated Radiology Reporting Template for Multiple Sclerosis Follow-Up. Proceedings of the 2020 European Congress of Radiology.

[35] Sima D.M., Jain S., Roura E., Maertens A., Smeets D., Sappey-Marinier D., Durand-Dubief F., Van Hecke W. New and Enlarging Lesion Location for Different MS Clinical Phenotypes. Proceedings of the MSParis2017—7th Joint ECTRIMS-ACTRIMS.

[36] Potemkowski A., Brola W., Ratajczak A., Ratajczak M., Zaborski J., Jasińska E., Pokryszko-Dragan A., Gruszka E., Dubik-Jezierzańska M., Podlecka-Piętowska A. (2019). Internet Usage by Polish Patients with Multiple Sclerosis: A Multicenter Questionnaire Study. Interact. J. Med. Res..

[37] Brand J., Köpke S., Kasper J., Rahn A., Backhus I., Poettgen J., Stellmann J.-P., Siemonsen S., Heesen C. (2014). Magnetic Resonance Imaging in Multiple Sclerosis--Patients’ Experiences, Information Interests and Responses to an Education Programme. PLoS ONE.

[38] Ruano L., Portaccio E., Goretti B., Niccolai C., Severo M., Patti F., Cilia S., Gallo P., Grossi P., Ghezzi A. (2017). Age and Disability Drive Cognitive Impairment in Multiple Sclerosis across Disease Subtypes. Mult. Scler..

[39] Nijeholt G.J., van Walderveen M.A., Castelijns J.A., van Waesberghe J.H., Polman C., Scheltens P., Rosier P.F., Jongen P.J., Barkhof F. (1998). Brain and Spinal Cord Abnormalities in Multiple Sclerosis. Correlation between MRI Parameters, Clinical Subtypes and Symptoms. Brain.

[40] Vercruyssen S., Brys A., Verheijen M., Steach B., Van Vlierberghe E., Sima D.M., Smeets D. (2020). Conformance to CMSC Magnetic Resonance Imaging (MRI) Guidelines in a Real-World Multicenter MRI Dataset. Int. J. MS Care.

[41] Tsagkas C., Magon S., Gaetano L., Pezold S., Naegelin Y., Amann M., Stippich C., Cattin P., Wuerfel J., Bieri O. (2019). Preferential Spinal Cord Volume Loss in Primary Progressive Multiple Sclerosis. Mult. Scler. J..

[42] Dahan A., Wang W., Gaillard F. (2018). Computer-Aided Detection Can Bridge the Skill Gap in Multiple Sclerosis Monitoring. J. Am. Coll. Radiol..

[43] Wang W., van Heerden J., Tacey M.A., Gaillard F. (2017). Neuroradiologists Compared with Non-Neuroradiologists in the Detection of New Multiple Sclerosis Plaques. AJNR Am. J. Neuroradiol..

[44] van Heerden J., Rawlinson D., Zhang A.M., Chakravorty R., Tacey M.A., Desmond P.M., Gaillard F. (2015). Improving Multiple Sclerosis Plaque Detection Using a Semiautomated Assistive Approach. AJNR Am. J. Neuroradiol..

[45] Zopfs D., Laukamp K.R., Paquet S., Lennartz S., Pinto Dos Santos D., Kabbasch C., Bunck A., Schlamann M., Borggrefe J. (2019). Follow-up MRI in Multiple Sclerosis Patients: Automated Co-Registration and Lesion Color-Coding Improves Diagnostic Accuracy and Reduces Reading Time. Eur. Radiol..

[46] Beadnall H.N., Ly L., Wang C., Billiet T., Ribbens A., Van Hecke W., Zivadinov R., Barnett M.H. (2018). 103 Exploring the Influence of Quantitative Magnetic Resonance Imaging on Decision-Making in Multiple Sclerosis Clinical Practice. J. Neurol. Neurosurg. Psychiatry.

[47] Lexa F.J. (2021). Duty Hour Limits for Radiologists: It’s About Time. J. Am. Coll. Radiol..

[48] Confavreux C., Vukusic S. (2006). Natural History of Multiple Sclerosis: A Unifying Concept. Brain.

